# Ventilation of Sleeping Apartments

**Published:** 1864-08

**Authors:** 


### Selections.

VENTILATION OF SLEEPING APPARTMENTS.—Mr. W. C. Burder writes the following on the ventilation of sleeping apartments; "On an average, I suppose, one third of a man's life is spent in a sleeping room and in such room he breathes an atmosphere which, in the majority of cases, even among the middle and upper classes, is a poor supporter of life. If he be robust, he may not be able to detect the effect of the indifferent air, but it is very likely that where a man under ordinary circumstances now lives to the age of seventy, with pure air all his life in his bed room he would live to the age of eighty. To those who are not robust the difference between pure and impure air, for a third part of life, is of vital importance, and turns the scale. To change the air of a room at night, and to introduce fresh air from without, must be attended with a fall of temperature which even the robust do not relish, and those who are not robust can not possibly endure, unless vigorous mechanical means are adopted to supply the heat which is abstracted by the introduction of the cold external air. Looking at the subject in this light, it occurred to me to try the plan of partial ventilation. A sleeper remaining in one part of the room does not require that the whole room shall be ventilated, I soon found that on the partial ventilation principle the great temperature difficulty could be overcome in a very simple manner, and in a most inexpensive one. If a tube which has direct communication with the external air, and its internal opening, near the sleeper, be sufficiently long and thin, either in coil or elongated, to allow the air in passing through to attain the same temperature as the average air of the room, a person lying in bed may breathe pure air without suffering from draught, and, indeed, he may so manage the simple machinery by which the air is conveyed to him, that he may only perceive a delightful freshness in the air. I have tried the plan and found it answers.—*Medical and Surgical Reporter.*

